# Prenatal Choline Attenuates the Elevated Adiposity and Glucose Intolerance Caused by Prenatal Alcohol Exposure

**DOI:** 10.3390/cells14181429

**Published:** 2025-09-12

**Authors:** Susan M. Smith, Carolyn A. Munson, George R. Flentke, Sandra M. Mooney

**Affiliations:** 1 Nutrition Research Institute, University of North Carolina at Chapel Hill, Kannapolis, NC 28081, USA; 2 Department of Nutrition, University of North Carolina at Chapel Hill, Kannapolis, NC 28081, USA

**Keywords:** Fetal Alcohol Spectrum Disorder, obesity, diabetes, metabolic syndrome, developmental origins of health and disease

## Abstract

Prenatal alcohol exposure (PAE) causes neurobehavioral deficits and metabolic syndrome in later life. Prenatal choline supplementation (PCS) improves those behavioral deficits. Here we test whether PCS also ameliorates the attendant metabolic syndrome, using an established mouse model that mirrors aspects of alcohol-related neurodevelopmental disorders. Pregnant dams were exposed to alcohol (3 g/kg) from gestational days 8.5–17.5; some dams received additional choline (175% of requirement) by a daily injection. Offspring were followed through to the age of 86 wks with respect to their body composition and glucose tolerance. We found that PAE affected these outcomes in a sex-dependent manner. Male PAE offspring exhibited an increased fat mass, liver enlargement, elevated fasting glucose, and glucose intolerance. Female PAE offspring exhibited an increased fat mass, but the glucose tolerance and fasting values were unaffected. Regardless of sex, PCS attenuated all these metabolic measures. PCS was shown previously to elevate methyl-related choline metabolites and improve fetal growth, suggesting that it acts by attenuating the in utero stressors that otherwise program the fetus for metabolic syndrome in later life. Importantly, PCS also improved the adiposity, fasting glucose, and glucose tolerance in control offspring consuming the fixed-nutrient AIN-93G diet, suggesting that its choline content (1 g/kg) may be inadequate for optimal rodent health.

## 1. Introduction

Prenatal alcohol exposure (PAE) is the most common, preventable teratogen exposure in many countries. Its most salient features include persistent behavioral and cognitive deficits, early growth deficits, and dysmorphologies that may include a distinctive craniofacial appearance that often initiates a diagnosis [1]. There is growing awareness that PAE also increases the risk for chronic disease onset in later life. A unique, participant-run survey of adults with PAE identified increased risks for hypertension, diabetes, and immune-related disorders, among other concerns [2]. These higher rates of obesity/overweight, diabetes, hyperlipidemia, vascular dysfunction, and hypertension are further documented in clinical cohorts of affected individuals [3,4,5,6,7,8,9]. Preclinical models affirm that these disorders are directly attributable to PAE, as those offspring display increased adiposity, worsened glucose tolerance, elevated blood pressure, and vascular dysfunctions at adulthood [9,10,11,12,13,14,15,16,17]. Alcohol use during pregnancy is common and its rates exceed 30% in Western nations including Australia, Russia, Brazil, and much of Europe [18]. In the U.S., 3.8% of pregnant women admit to binge drinking (defined as more than three drinks per occasion) in the prior month, and 13.6% self-report any alcohol consumption [19]. Thus, PAE is an under-recognized and significant contributor to the high rates of chronic disease and metabolic dysfunction in these populations.

PAE likely affects adult health as an in utero stressor that adversely affects fetal and placental development [20]. High exposures reduce placental and fetal growth, whereas lower exposures reshape the placental size and efficiency in a sex-specific manner [21,22,23]. PAE also impairs placental vascularization and remodeling to limit blood flow and nutrient availability, and this imposes additional stress [23,24,25,26]. Such in utero stressors could reprogram the fetus, perhaps via epigenetic mechanisms, to anticipate similar nutrient stressors in later life [27]. However, under postnatal environments of nutrient adequacy in later life, this instead increases the offspring’s risk of metabolic disorders, a phenomenon known as the developmental origins of adult disease, or DOHaD [28,29,30]. Numerous studies of human populations that experience in utero stress, as well as preclinical models of such stress, document that these increase the offspring’s risk for overweight and obesity, type-2 diabetes, and hypertension in later life [28,29,30].

Given the high incidence of PAE and its severe behavioral and physiological consequences, there is a great interest in interventions that attenuate its effects. One of these is the essential micronutrient choline and good clinical and preclinical evidence documents that prenatal choline supplementation (PCS) improves brain development and cognitive and behavioral outcomes in response to PAE [31,32,33,34]. It is possible that PCS might also reduce the risk for metabolic dysfunction in PAE offspring because it also improves those perinatal measures that are indicative of in utero stress, including fetal and infant weight, infant growth, head circumference, and placental efficiency [32,33,34,35,36]. Here, we show that prenatal choline supplementation acts in a sex-dependent manner to reduce adiposity and improve glucose tolerance in aged mice that experienced PAE.

## 2. Materials and Methods

### 2.1. Mice

Female and male C57BL6/J mice were purchased from Jackson Laboratories (Bar Harbor, ME, USA) at age 5 weeks. At age 8–9 weeks, they were mated overnight; the morning of vaginal plug detection was defined as gestational day (GD) 0.5. Offspring were weaned at postnatal day (P) 22 and housed in sex-matched groups of 4–5 animals. Offspring were weighed routinely. Throughout this study, breeding females and all offspring consumed a nutritionally adequate, fixed-nutrient diet, AIN-93G (Envigo Teklad, Madison, WI, USA) [37]. This diet provides 18.8% kcals as protein, 17.2% kcals as fat, and 63.9% kcals as carbohydrate and is considered moderate with respect to its sugar and fat content. Mice were phenotyped as described below at ages 3 mo, 9 mo, 13 mo, and 19 mo. Animals were euthanized at age 86 wks. All protocols were approved by the Animal Care and Use Committee for the North Carolina Research Campus.

### 2.2. Alcohol Exposure and Choline Intervention

Pregnant dams were assigned to exposure (±PAE) and intervention (±PCS) using a random-number generator. Pregnant female mice received either alcohol (ALC; USP grade, 200 proof; Koptek, King of Prussia, PA, USA) or maltodextrin (CON; LoDex-10, Envigo Teklad) daily by oral gavage from GD8.5 through GD17.5. The alcohol dose was 3 g/kg body weight and generated blood alcohol levels of 211 ± 14 at 30 min after dosing [38].

For the prenatal choline intervention, dams received a subcutaneous injection of choline chloride (100 mg/kg; Balchem, New Hampton, NY, USA) once daily immediately after the oral gavage from GD8.5 through GD17.5 [35]. This choline dose provides 74.6 mg free choline/kg body weight and represents an additional 75% choline above that delivered by the AIN-93G diet.

### 2.3. Body Composition

Body composition was determined at ages 13 mo and 19 mo using an MRI scanner (EchoMRI-100H, Houston, TX, USA). Mice were anesthetized under isoflurane prior to being scanned. Lean and fat mass were quantified and percentage lean and fat mass were calculated from body weight taken just prior to the scan.

### 2.4. Glucose Tolerance Testing

This was performed 1–2 days prior to body composition analysis at ages 13 mo and 19 mo. Mice were fasted for five hours by placing them in a fresh cage with no food and with free access to water [39]. A blood drop was taken from tail snip and fasting blood glucose was immediately quantified using a hand-held glucometer (OneTouch Ultra 2; LifeScan Inc., Milpitas, CA, USA). Glucose (10% in 1× phosphate buffered saline; USP grade, Sigma-Aldrich, St. Louis, MO, USA) was administered by intraperitoneal injection at a dose of 1.0 g/kg body weight. Blood glucose was quantified at 15, 30, 60, 90, and 120 min post-injection. Glucose clearance was quantified using the trapezoidal rule to calculate the area-under-the-curve (AUC) for both the original values and values normalized to each animal’s fasting blood glucose level.

### 2.5. Statistical Analysis

Data were tested for normality using the Shapiro–Wilk test and for equal variance using the Brown–Forsythe test. Data on body weights over time were analyzed using a linear mixed model with sex, age, alcohol exposure, and choline treatment as factors; this was chosen over repeated measures as not all animals were present at all timepoints. Where interactions with age were identified, linear regression analyses were run to understand the contribution of age within that factor. Body weight affects organ weight and body composition, and body composition affects glucose tolerance. As the linear mixed model identified effects of age and sex on body weight, the analyses of organ weight, body composition, and glucose metabolism were stratified by age and sex and analyzed using two-way ANOVA with alcohol exposure and choline intervention as factors. Significant outcomes were further analyzed using the Holm–Sidak pairwise multiple comparisons procedure to identify between-group differences. Significance was assigned at *p* ≤ 0.05 and trends are reported where 0.051 < *p* < 0.1. Analysis was performed using SigmaPlot (ver. 12, Systat Software, Inc., San Jose, CA, USA) or SPSS (v.31, IBM, Armonk NY, USA). For each outcome, the effects of prenatal alcohol exposure are first presented, followed by the effects of choline intervention.

## 3. Results

### 3.1. PAE and Choline Affect Body Weight Gains and Brain and Hepatic Weight in Aged Male and Female Mice

**Figure 1:**
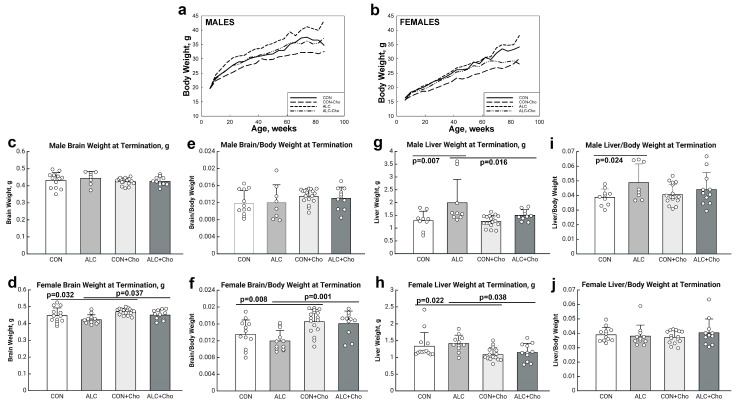
Growth and somatic measures. (**a**,**b**) Body growth of males (**a**) and females (**b**) from age 6 wks to 86 wks. Data are mean values and variance is omitted for clarity. (**c**,**d**) Male (**c**) and female (**d**) brain weight at study termination of 86 wks. (**e**,**f**) Male (**e**) and female (**f**) brain weight normalized to body weight at 86 wks. (**g**,**h**) Male (**g**) and female (**h**) liver weight at 86 wks. (**i**,**j**) Male (**i**) and female (**j**) liver weight normalized to body weight at 86 wks. (**c**–**h**) depict mean ± SD; symbols represent individual animals. Abbreviations: CON, control; ALC, alcohol-treated; CON + Cho, controls + prenatal choline supplement; ALC + Cho, alcohol-treated + prenatal choline supplement.

We found main effects of sex (F_1,2808_ = 835.5, *p* < 0.001), age (F_28,2808_ = 121.1, *p* < 0.001), alcohol exposure (F_1,2808_ = 114.4, *p* < 0.001), and choline treatment (F_1,2808_ = 283.4, *p* = 0.050) on offspring growth. There were also interactions of sex and alcohol (F_1,2808_ = 16.48, *p* < 0.001), sex and age (F_19,191_ = 7.379, *p* < 0.001), alcohol and age (F_19,191_ = 3.597, *p* < 0.001), and treatment and age (F_19,191_ = 4.859, *p* < 0.001). The sex × alcohol interaction showed that ALC males were heavier than CON males (Figure 1a) and ALC females were heavier than CON females (Figure 1b). Linear regressions were also used to understand the interactions with age. The sex and age interaction identified that males were heavier than females at all ages and that females gained more weight with age than males (male weight = 22.778 g + (0.185 g × age) and female weight = 16.014 g + (0.200 g × age)). The alcohol × age interaction showed that ALC mice were significantly heavier than CON between 18 and 78 weeks of age and that ALC mice gained more weight with age than CON (CON weight = 19.129 g + (0.176 g × age) and ALC weight = 19.662 g + (0.209 g × age)). The interaction between age and choline treatment showed that from age 10 wk onwards, the choline-treated animals had lower body weights than the untreated animals and they gained less weight with age than the untreated mice (no choline weight = 19.417 g + (0.222 g × age) and choline-treated weight = 19.374 g + (0.163 g × age)). When analyzed by sex, the treatment × age interaction was strongest in females, and the choline-treated females were lighter than no-choline females, irrespective of the CON or ALC status, from age 38 wk onwards. The average weight difference between 38 and 86 wk was 4.3 g. For the males, the weight difference due to choline emerged later, at age 62 wk, and the average weight difference between 62- and 86 wk was 4.0 g. In summary, PAE increased the body weight of both ALC males and ALC females. The prenatal choline intervention reduced their body weights to levels that were equivalent to and sometimes lower than CON-no choline. The choline effect was stronger in ALC + Cho females than ALC + Cho males. Additionally, the intervention also reduced the body weights of CON + Cho animals to values below those of CON-no choline, and this was present for both sexes.

The late-term ALC fetuses generated in this PAE model have a consistent, 5% reduction in body weight, reduced liver weight, and normal brain weight, indicating a brain-sparing effect [35,38]. We assessed the long-term impact of PAE on these measures, assessed at study termination (age 86 wks). Because body weight affects the organ weight and the males were heavier than females, we analyzed the sexes separately. Neither PAE nor choline affected the adult brain weight in males. However, both alcohol exposure (F_1,48_ = 5.642, *p* = 0.022) and choline treatment (F_1,48_ = 9.439, *p* = 0.003) affected the female brain weight, and prenatal choline was associated with a 5% increase in brain weight for both CON (*p* = 0.032) and ALC (*p* = 0.037) females (Figure 1c–f). Choline (F_1,45_ = 10.147, *p* = 0.003), but not alcohol, also affected the female liver weight and both CON (*p* = 0.022) and ALC (*p* = 0.038) livers were 22.7% to 24.2% heavier than those receiving prenatal choline (Figure 1g–j). In the males, there were effects of both PAE (F_1,42_ = 7.602, *p* = 0.009) and choline (F_1,42_ = 4.637, *p* = 0.037) upon the liver. Male ALC livers were 46.7% heavier than CON livers (*p* = 0.007), and choline ameliorated (*p* = 0.016) this effect of PAE. Choline did not affect the liver weight in CON males.

### 3.2. PAE Increases Adiposity in Male and Female Mice

To understand the basis for these body weight differences, we evaluated their body composition at ages 13 mo and 19 mo. Because sex and age affected body composition, these were analyzed separately. Pandemic restrictions prevented their full assessment at ages 3 mo and 9 mo, and thus, those data are not reported here. However, the growth curves at those ages did not indicate alcohol- or choline-mediated differences, suggesting their body composition was also unaffected.

**Figure 2:**
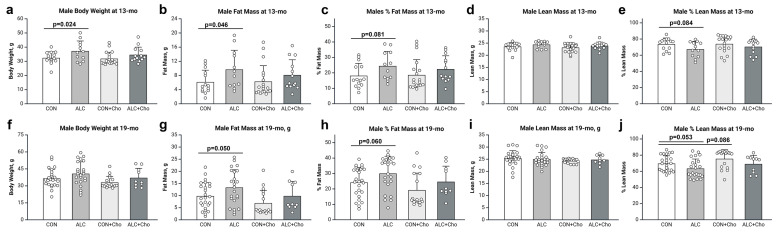
Body composition of males at ages 13 mo (**a**–**e**) and 1–9 mo (**f**–**j**). (**a**,**f**) Body weight. (**b**,**g**) Fat mass as determined by MRI. (**c**,**h**) Percentage fat mass. (**d**,**i**) Lean mass as determined by MRI. (**e**,**j**) Percentage lean mass. Values are mean ± SD; symbols represent individual animals. Abbreviations: CON, control; ALC, alcohol-treated; CON + Cho, controls + prenatal choline supplement; ALC + Cho, alcohol-treated + prenatal choline supplement.

PAE had sex-dependent effects on the body weight and composition. At age 13 mo, there was an alcohol effect in males (F_1,57_ = 7.216, *p* = 0.010), and untreated ALC mice were heavier than untreated CON (CON, 32.31 ± 1.41 g; ALC, 37.13 ± 1.52 g; *p* = 0.024) (Figure 2a). This represented PAE effects on the absolute fat mass (F_1,57_ =4.064, *p* = 0.049), % fat mass (F_1,57_ = 4.397, *p* = 0.041), and % lean mass (F_1,57_ = 4.064, *p* = 0.049). PAE males had more fat mass (CON, 6.11 ± 1.18 g; ALC, 9.65 ± 1.27 g; *p* = 0.046; Figure 2b) and trended to both a greater % adiposity (CON, 18.05 ± 2.42%; ALC, 24.38 ± 2.62%; *p* = 0.081; Figure 2c) and reduced % lean mass (CON,73.60 ± 2.47%; ALC, 67.20 ± 2.67%; *p* = 0.084; Figure 2e). PAE did not affect the absolute lean mass (Figure 2d), indicating that these compositional differences were driven by increased adiposity.

These differences persisted at age 19 mo. PAE again affected male body weight (F_1,74_ = 3.975, *p* = 0.050) and the untreated ALC males were somewhat heavier than age-matched untreated CON males (CON, 36.4 ± 1.69 g; ALC, 40.64 ± 1.76 g; Figure 2f). This weight difference again represented an effect of PAE upon absolute fat mass (F_1,74_ = 4.618, *p* = 0.035) and % fat mass (F_1,74_ = 4.730, *p* = 0.033). The untreated ALC males had a greater fat mass (CON, 8.30 ± 0.96 g; ALC, 13.28 ± 1.28 g; *p* = 0.050; Figure 2g) and trended to a greater % fat mass (CON 24.14 ± 2.10%; ALC, 29.94 ± 2.19%; *p* = 0.060; Figure 2h). Lean mass did not differ (Figure 2i,j).

**Figure 3:**
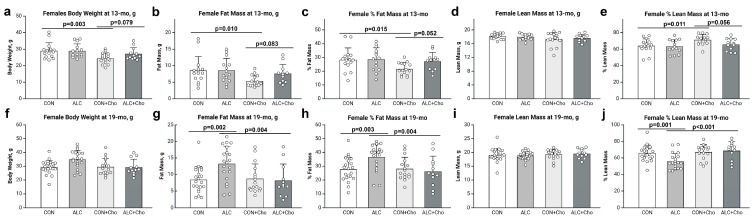
Body weight and composition of females at ages 13 mo (**a**–**e**) and 19 mo (**f**–**j**). Top row—age 13 mo. Bottom row—age 19 mo. (**a**,**f**) Body weight. (**b**,**g**) Fat mass as determined by MRI. (**c**,**h**) Percentage fat mass. (**d**,**i**) Lean mass as determined by MRI. (**e**,**j**) Percentage lean mass. Values are mean ± SD; symbols represent individual animals. Abbreviations: CON, control; ALC, alcohol-treated; CON + Cho, controls + prenatal choline supplement; ALC + Cho, alcohol-treated + prenatal choline supplement.

Females responded differently to PAE. At age 13 mo, body composition did not differ between ALC and CON females (Figure 3a–e). At age 19 mo, PAE trended to affect body weight (F_1,68_ = 3.431, *p* = 0.069) and fat mass (F_1,68_ =3.435, *p* = 0.068). Untreated ALC females had heavier body weights (CON, 29.21 ± 1.28 g; ALC, 34.75 ± 1.31 g; Figure 3f), representing gains in their absolute fat mass (CON, 8.38 ± 1.02 g; ALC, 13.20 ± 1.04 g; *p* = 0.002; Figure 3g) and % fat mass (CON, 27.53 ± 2.05%; ALC, 36.43 ± 2.10%; *p* = 0.003; Figure 3h). Their absolute lean mass did not differ, indicating similar postnatal growth (Figure 3i). However, their greater adiposity reduced their % lean mass (CON, 65.78 ± 2.12%; ALC, 55.58 ± 2.18%, *p* = 0.001; Figure 3j).

In summary, PAE increased the adiposity of both male and female offspring as compared with age-matched unexposed controls, and their adiposity worsened as the animals aged. PAE did not affect their absolute lean mass.

### 3.3. Prenatal Choline Attenuates PAE-Dependent Adiposity in Male and Female Mice

The prenatal choline intervention affected adiposity in an age-, sex-, and exposure-dependent manner, with commensurate shifts in % lean mass, but no effects on absolute lean mass. For the males, choline affected body weight and composition at 19 mo but not at 13 mo (Figure 2a–e). At 19 mo, choline weakly trended (F_1,74_ = 2.895, *p* = 0.093) to affect body weight, and both CON + Cho (CON, 36.4 ± 1.69 g; CON + Cho 32.95 ± 2.05 g) and ALC + Cho males (ALC, 40.64 ± 1.76 g; ALC + Cho, 37.02 ± 2.67 g) had slightly lower body weights than their no-choline counterparts (Figure 2f). The ALC + Cho males were still heavier than CON + Cho males. Prenatal choline had greater effects on body composition, affecting both fat mass (F_1,74_ = 4.525, *p* = 0.037; Figure 2g) and % fat mass (F_1,74_ = 4.036, *p* = 0.048; Figure 2h) to reduce the adiposity of both CON and ALC mice, although their differences did not achieve significance in the multiple comparisons. Choline also affected % lean mass (F_1,74_ = 4.758, *p* = 0.032) such that the CON + Cho and ALC + Cho males had a greater % lean mass than their non-choline counterparts (CON + Cho, 75.28 ± 2.62%; ALC + Cho, 68.91 ± 3.41%; Figure 2j). This represented the loss of adiposity because their absolute lean mass was unaffected (Figure 2i). Thus, in the 19 mo males, PAE increased their body weight and adiposity, and supplemental choline attenuated those effects of PAE. Choline also reduced the adiposity of the control males.

Choline differently affected the body composition of the females. These effects were manifested at younger ages to affect female body weight (F_1,52_ = 8.399, *p* = 0.005; Figure 3a), fat mass (F_1,52_ = 5.788, *p* = 0.020; Figure 3b), % fat mass (F_1,52_ = 4.376, *p* = 0.041; Figure 3c), and % lean mass (F_1,52_ = 5.684, *p* = 0.021; Figure 3e) at age 13 mo. However, at this age the prenatal choline only benefited the controls, and the CON-Cho females were lighter (CON, 28.91 ± 1.05 g; CON + Cho, 24.32 ± 1.05 g; *p* = 0.003), with a reduced fat mass (CON, 8.51 ± 0.84 g; CON + Cho, 5.33 ± 0.84 g; *p* = 0.010), % fat mass (CON, 28.18 ± 1.85%; CON + Cho, 21.58 ± 1.85%; *p* = 0.015), and % lean mass (CON, 63.93 ± 1.94%; CON + Cho, 71.17 ± 1.94%; *p* = 0.011). Similar to their male littermates, prenatal choline did not affect the body composition or adiposity of the 13 mo old ALC females. Choline did not affect the absolute lean mass in the CON or ALC mice (Figure 3i), indicating similar postnatal somatic growth.

The protective effect of choline upon the PAE females emerged at age 19 mo and was preserved in CON females at that age. Choline trended to affect body weight (F_1,65_ = 3.53, *p* = 0.065; Figure 3f) and affected fat mass (F_1,65_ = 4.330, *p* = 0.041; Figure 3g), % fat mass (F_1,65_ = 4.496, *p* = 0.038; Figure 3h), and % lean mass (F_1,65_ = 8.70, *p* = 0.004; Figure 3j) in these females. Choline also interacted with PAE to affect body weight (F_1,65_ = 3.972, *p* = 0.050), fat mass (F_1,65_ = 5.43, *p* = 0.023), and % lean mass (F_1,65_ = 6.09, *p* = 0.016). Specifically, choline reduced the body weight of PAE females such that it no longer differed from either CON or CON + Cho (CON + Cho, 29.38 ± 1.46 g; ALC + Cho, 29.17 ± 1.69 g) mice. This effect of prenatal choline represented a reduction in the absolute fat mass (ALC vs ALC + Cho, *p* = 0.002) and % fat mass (ALC vs ALC + Cho, *p* = 0.004) of the PAE females, and their adiposity did not differ from that of CON or CON + Cho females (CON + Cho, 8.66 ± 1.17 g; ALC + Cho, 8.11 ± 1.35 g). This loss of adiposity was further affirmed by the corresponding improvement in % lean mass (ALC vs ALC + Cho, *p* < 0.001) with no change in absolute lean mass (Figure 3i). Choline did not improve the adiposity of CON females at age 19 mo, indicating that choline’s action was selective for PAE at this age.

In summary, prenatal choline reversed the PAE-associated adiposity in male and female offspring at age 19 mo. It also improved the adiposity of CON females at age 13 mo and of CON males at age 19 mo. The lack of effect upon the absolute lean mass in either CON or ALC indicated that choline’s actions selectively targeted adiposity and not postnatal somatic growth.

### 3.4. PAE Worsens Glucose Tolerance in Male but Not Female Mice

Elevated adiposity may reflect impairments in the ability to metabolize glucose and PAE is known to worsen glucose tolerance at adulthood [7,8,9,10,11,12,13,14,15,16,17]. We assessed the influence of PAE and prenatal choline upon glucose metabolism using an intraperitoneal glucose tolerance test (IPGTT), which bypasses secondary influences of the enteric system. Because both age and sex affected body composition, and because body composition affects glucose tolerance, the data analyses were stratified by sex and age.

**Figure 4:**
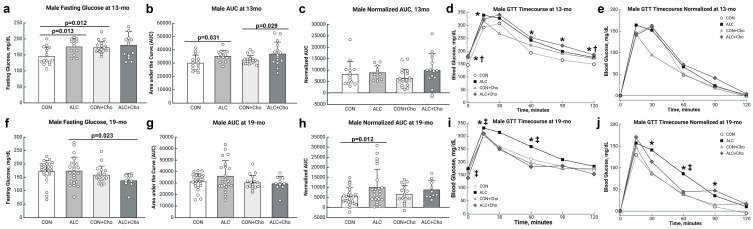
Blood glucose parameters in males at (**a**–**e**) 13 mo and (**f**–**j**) 19 mo. (**a**,**f**) Fasting blood glucose. (**b**,**g**) Blood glucose clearance evaluated as area-under-the-curve (AUC). (**c**,**h**) Blood glucose AUC normalized to fasting glucose values. (**d**,**i**) Timecourse of blood glucose clearance. (**e**,**j**) Timecourse of blood glucose clearance normalized to fasting glucose. * ALC differs from CON at *p* < 0.05. † CON differs from CON + Cho at *p* < 0.05. ‡ ALC differs from ALC + Cho at *p* < 0.05. In (**a**–**c**,**f**–**h**), values are mean ± SD; symbols represent individual animals. (**d**,**e**,**i**,**j**) are mean values with variance omitted for clarity. Abbreviations: CON, control; ALC, alcohol-treated; CON + Cho, controls + prenatal choline supplement; ALC + Cho, alcohol-treated + prenatal choline supplement.

We found age-, sex-, and exposure-specific effects on glucose tolerance. In 13 mo male mice, PAE affected fasting glucose (F_1,56_ = 5.930, *p* = 0.018) and glucose clearance (F_1,56_ = 9.867, *p* = 0.003). ALC males had a higher level of fasting glucose (CON, 146 ± 8 mg/dL; ALC, 175 ± 9 mg/dL; *p* = 0.013; Figure 4a) and elevated AUCs in the IPGTT (*p* = 0.031; Figure 4b,d). By age 19 mo, fasting blood glucose levels in CON had risen and no longer differed from those of PAE mice (Figure 4f), nor did their absolute AUC in the IPGTT (Figure 4g,i). The normalization of AUC to the animal’s baseline (fasting) glucose level clarifies the ability to clear the glucose challenge [39]. This revealed that PAE affected glucose clearance (F_1,71_ =5.184, *p* = 0.026), such that aged ALC by 19 mo had worsened the glucose clearance compared with CON (CON, 5374 ± 1235; ALC, 9975 ± 1287; *p* = 0.012; Figure 4h,j). Although both ALC and CON mice achieved similar peak blood glucose values at 15 min following the glucose injection, the CON males had improved glucose clearance at times thereafter, as reflected in their lower blood glucose values at 30 min (*p* = 0.014) and 90 min (*p* = 0.041) and a trend to reduction at 60 min (*p* = 0.070) post-injection.

**Figure 5:**
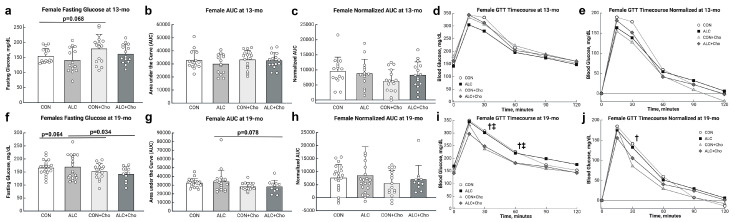
Blood glucose parameters in females at (**a**–**e**) 13 mo and (**f**–**j**) 19 mo. (**a**,**f**) Fasting blood glucose. (**b**,**g**) Blood glucose clearance evaluated as area-under-the-curve (AUC). (**c**,**h**) Blood glucose AUC normalized to fasting glucose values. (**d**,**i**) Timecourse of blood glucose clearance. (**e**,**j**) Timecourse of blood glucose clearance normalized to fasting glucose. † CON differs from CON + Cho at *p* < 0.05. ‡ ALC differs from ALC + Cho at *p* < 0.05. Values in (**a**–**c**,**f**–**h**) are mean ± SD; symbols represent individual animals. (**d**,**e**,**i**,**j**) present mean values with variance omitted for clarity. Abbreviations: CON, control; ALC, alcohol-treated; CON + Cho, controls + prenatal choline supplement; ALC + Cho, alcohol-treated + prenatal choline supplement.

In contrast, PAE did not affect glucose tolerance in the female offspring. At ages 13 mo and 19 mo, the ALC and CON females had similar fasting glucose levels (Figure 5a,f) and similar abilities to clear the glucose load, as reflected in AUC or normalized AUC (Figure 5b–e,g–j).

### 3.5. Prenatal Choline Improves Glucose Tolerance in Male and Female PAE Mice

The prenatal choline intervention affected glucose tolerance in a sex-, age-, and exposure-dependent manner. In the 13 mo old males, choline was associated (F_1,56_ = 4.313, *p* = 0.042) with elevated fasting glucose in CON (*p* = 0.012; Figure 4a), but not in ALC and did not affect glucose clearance in either group (Figure 4b–e). However, by age 19 mo, choline affected the fasting blood glucose (F_1,71_ = 5.907, *p* = 0.018) such that ALC + Cho males had a lower level of fasting glucose than ALC males who did not receive choline (ALC, 173 ± 9 mg/dL, ALC + Cho, 138 ± 13 mg/dL; *p* = 0.023; Figure 4f). Although choline did not affect the peak blood glucose, AUC, or normalized AUC values during the IPGTT in ALC mice (Figure 4g–i), it improved their ability to clear the glucose load, as reflected in lower blood glucose values at 30 min (*p* = 0.046) and 60 min (*p* = 0.008) following the challenge (Figure 4i). Choline did not affect fasting glucose or glucose clearance in the 19 mo old CON males.

In the females at 13 mo, choline affected fasting glucose (F_1,56_ = 5.387, *p* = 0.024), and this represented a trend for CON + Cho to have higher fasting glucose than CON (CON, 154 ± 10 mg/dL; CON + Cho, 179 ± 10 mg/dL; *p* = 0.068; Figure 5a), similar to the response in CON males at this age. Choline did not affect glucose clearance in either CON or PAE females at 13 mo (Figure 5b–e). However, similar to the males, by age 19 mo, choline again affected fasting blood glucose (F_1,60_ = 8.269, *p* = 0.006) and glucose clearance (F_1,60_ = 5.653, *p* = 0.021). Choline reduced the fasting blood glucose in ALC females (ALC, 168 ± 7 mg/dL; ALC + Cho, 141 ± 10 mg/dL; *p* = 0.034) and trended to reduce it in CON (CON, 174 ± 8 mg/dL; CON + Cho, 151 ± 8 mg/dL; *p* = 0.064; Figure 5f). Prenatal choline also trended to improve the ability of PAE females to clear the glucose challenge, and ALC + Cho females had somewhat lower AUCs than their PAE counterparts (ALC, 33940 ± 1927; ALC + Cho, 28230 ± 2599; *p* = 0.078; Figure 5g,i). Although the choline intervention did not reduce the absolute AUC in CON females, it trended to reduce the blood glucose at 15 min (CON, 349 ± 16 mg/dL; CON + Cho, 330 ± 18 mg/dL; *p* = 0.077) and 60 min (CON, 244 ± 15 mg/dL; CON + Cho, 180 ± 17 mg/dL; *p* = 0.053) and reduced the blood glucose at 30 min (CON, 307 ± 20 mg/dL; CON + Cho, 236 ± 23 mg/dL; *p* = 0.023), suggesting their glucose tolerance was also improved. Normalized AUC values were unaffected by the choline intervention in CON and PAE females (Figure 5h,j).

## 4. Discussion

Both preclinical studies and clinical intervention trials show that prenatal choline supplementation (PCS) improves the birth weight, postnatal growth, and behavioral and cognitive outcomes in the offspring born to alcohol-exposed pregnancies [31,32,33,34,35,36]. Here, we extend its benefits to the metabolic dysfunctions that also are associated with PAE. Using a PAE model that mirrors aspects of alcohol-related neurodevelopmental disorders [38], we find that PCS ameliorates the elevated adiposity and glucose intolerance caused by PAE. Although PAE affected the glucose tolerance and adiposity in a sex-dependent manner, consistent with prior clinical and preclinical studies, the benefits of PCS extended to both sexes. These findings are summarized in Table 1. As PCS also improves measures of fetal growth and placental function in alcohol-exposed pregnancies [31,32,33,34,35,36], we speculate that PCS confers its benefits, at least in part, by reducing the in utero stress that would otherwise program the offspring for metabolic dysfunction in later life [28,29,30].

The effects of PAE upon metabolic dysfunction were a function of the offspring sex. Females trended to exhibit a greater and earlier adiposity than did their male littermates (345% vs 220%), although this sex difference was not as striking as in prior work in this model [16]. In contrast, males had a worsened glucose intolerance that was largely absent from the females. These outcomes are similar to prior work in this model [16] and to findings in children and adolescents [6,40,41] and adults diagnosed with FASD [9]. In the adult subjects [9], males were underweight with lower BMIs yet had higher rates of type-2 diabetes mellitus (T2DM). In contrast, females had a risk for T2DM that was commensurate with their elevated BMIs, suggesting that their respective dysfunctions may have had different origins. Greater female adiposity is also observed in guinea pig [11], mouse [17,42], and rat models of PAE [13,43], suggesting this may be a consistent consequence of PAE. Insight into potential mechanisms comes from observations that the moderate exposure used here causes a consistent but non-significant 5% reduction in fetal body weight at late term [34,38]. It also reduces the fetal liver and brain weight, both absolute and normalized to body weight, and this effect of PAE is stronger in males than in females [35]. Intrauterine growth restriction is an independent risk factor for obesity and T2DM in later life [44], perhaps due to epigenetic adaptations within the fetus that reduce growth and facilitate survival in a nutrient-limiting uterine environment, but are mismatched with respect to nutritional adequacy in later life [28,29,30,44]. That this model produces fairly subtle intrauterine growth deficits may explain why these metabolic phenotypes only emerged in later life. It further suggests that stronger in utero stressors may lead to their manifestation at younger ages. The sexes utilize different adaptive strategies in response to in utero stress, such that female fetuses have greater growth reductions than males [45,46], and perhaps reflecting that IGF2, a major driver of intrauterine growth, is subject to paternal imprinting [47]. Although data are equivocal on whether PAE alters the imprinting of fetal growth genes, including the *IGF2*/*H19* locus [48,49], such imprinting could further account for the differential metabolic consequences of PAE.

The strength of these in utero adaptations may also explain why metabolic disorders are inconsistently observed in response to PAE. Some preclinical PAE models, including an early study from these authors, find no or weak effects on adiposity or glucose handling [14,15,42,50]. As per above, this may partly reflect the severity of fetal growth restriction, as well as differences in the timing of exposure, strain, control comparator (sucrose vs maltodextrin vs water), and postnatal diet. However, a closer review reveals that the null findings were more likely to result from assessment at young adulthood, at ages 12 to 20 weeks old and a time when somatic growth has only recently plateaued. Studies that instead focused on mature animals, age 6 months and older, are more likely to detect PAE-related metabolic deficits, and several longitudinal studies of PAE confirm their emergence with aging [14,15,16,17]. This suggests that the energy demands of growth at younger ages may mask or compensate for pre-existing metabolic deficits that create the physical phenotype once somatic growth has ceased. Many chronic diseases are the consequence of subtle, cellular-level changes—that is, shifts in activity rather than an absolute loss—that gradually accumulate to impair, for example, pancreatic, hepatic, or adipose function. Several factors that would magnify the risk for adiposity have been reported in PAE, including increased disordered eating [40], reduced spontaneous activity [9,42], and expression shifts in the gene networks modulating carbohydrate and lipid metabolism in some PAE models [9,13,51,52,53], but not in others [14,15]. However, PAE does not appear to affect metabolic activity per se, as measured using indirect calorimetry to quantify the respiratory exchange ratio [42,50], nor did it affect the animal’s ability to shift usage between carbohydrate and triglyceride-rich fuels [50]. However, PAE female mice had reduced fat oxidation during the sleep (light) cycle [42], which might indicate an increased food/carbohydrate intake during that period, circadian shifting [54,55], or impaired lipid catabolism and beta-oxidation [9], all of which could lead to greater adiposity. Although we found no PAE-related shifts in food intake at age 4 months [50], subtle shifts amounting to a few calories daily can produce steadily increasing adiposity [56] and may explain this phenotype in the PAE females.

Insight into the worsened glucose tolerance in PAE males emerges from preclinical models, which identify multiple sites of dysfunction. Male rats with PAE have an insulin-resistant, pre-diabetic phenotype with fasting elevated insulin and HOMA-IR and an elevated first-phase release of insulin [14,15], suggesting compensatory attempts to regulate blood glucose. The endocrine pancreas itself may be dysfunctional. The pancreas of a guinea pig model of PAE exhibits increased adipose infiltration [11], a pathology linked in humans to worsened glucose sensitivity [57]. These pancreata also had reduced exocrine tissue and fewer β-cells per islet, which could limit insulin production. Peripheral insulin clearance may also be impaired. PAE reduced the hepatic expression of insulin signaling components in males, but not in females [14,51]. PAE livers also exhibit an elevated expression of the gluconeogenic enzymes phosphoenolpyruvate carboxykinase (PEPCK) and glucose-6-phosphatase, which may have fetal origins [52], and these expression shifts are resistant to insulin-mediated suppression [13,53]. Such a failure to suppress glucose production would further elevate plasma glucose and suggests that the PAE liver is insulin-resistant. Elevations of activated pAKT(S473) in the white adipose tissue of PAE males may represent a compensatory attempt to enhance peripheral glucose clearance [13]. Less clear is why males are more vulnerable to PAE-associated insulin resistance than their female littermates, and this merits additional study. The alcohol exposure of mice in the neonatal period alters signaling within the pro-opiomelanocortin neurons of the arcuate nucleus [55], a brain region that also regulates feeding, and such disruptions could modulate appetitive signals in the PAE offspring.

How might PCS attenuate PAE-induced metabolic dysfunction? In otherwise normal pregnancies, PCS improves offspring growth, lean mass, and insulin sensitivity while reducing adiposity [58,59,60,61]. The elevation of choline and betaine via a loss-of-function in betaine hydroxymethyltransferase (*Bhmt*) confers similar benefits [62]. These actions of PCS are mechanistically complex. Epigenetic changes are reported for metabolic effectors involved in insulin signaling (*Irs1*, *Irs2*) [62], cortisol signaling (*Crh*, *Nr3c1*) [63], and food intake (*Kl*, *Crh*) [64]. Offspring exhibit increased glucose oxidation and reduced lipogenesis, perhaps in response to elevated PPARγ [58,62]. FGF21 is also elevated, perhaps via bile acids and/or endoplasmic reticulum stress, to promote adipose browning [62,65]. Selective elevations in choline-dependent plasmalogen phospholipids and sphingolipids may reflect a hepatic environment that is better adapted to accommodate metabolic stress [66]. Thus, while PCS is associated with a reduced risk for metabolic syndrome, the underlying mechanisms are multifactorial and incompletely understood.

Such mechanisms likely apply to PCS in the context of PAE. Alcohol increases hepatic lipogenesis [67] and the demand for phosphatidylcholine [68]. This includes the pregnant dam [52], and under PAE, her liver is characterized by elevated phosphatidylcholine and metabolites within the CDP-choline pathway by which choline enters those lipid pools [69]. Choline’s diversion into phospholipids comes at the expense of its other methylation-related fates, as seen in commensurate reductions in downstream metabolites including dimethylglycine and SAM/SAH ratios [69,70], reductions that may set up the fetus for metabolic syndrome in later life. PCS elevates maternal–fetal betaine, SAM, and SAM/SAH under PAE [69,70], indicating that PAE causes a functional choline insufficiency with respect to its methylation and/or epigenetic activities. Its remediation by PCS normalizes fetal growth and improves placental function, indicating that it attenuates the in utero stressors that otherwise drive metabolic disorders in later life [35,36,71].

Linked to this choline insufficiency is the hepatic enlargement observed in the aged PAE males. Their mean liver weights were nearly 50% heavier than in controls and, notably, this emerged under a diet with a moderate fat content. Given these animals’ adiposity and glucose intolerance, we speculate that this might represent metabolic dysfunction-associated steatotic liver disease (MASLD). Choline insufficiency is partly characterized by a hepatic steatosis that reflects insufficient phosphatidylcholine for hepatic lipid export [68]. PCS reduced hepatic weight in both PAE males and females, endorsing that PAE created a choline-limiting environment that heightens their vulnerability to the disorder. An increased risk for spontaneous MASLD has not been previously linked with PAE, but is consistent with recent findings that PCS protected PAE offspring from alcoholic fatty liver when they were exposed as young adults [72]. PAE alters hepatic morphogenesis [9] and leads to lobular inflammation and microvesicular steatosis [12] that could predispose for MASLD and alcoholic liver disease in later life. Collectively, these findings suggest that both PAE and prenatal choline insufficiency may be underappreciated contributors to MASLD [68], which affects 38% of adults and 7–14% of children and adolescents in the US [73].

Lastly, an unexpected finding was that the benefits of PCS with respect to adiposity and glucose tolerance extended to the offspring from control pregnancies that were not exposed to alcohol. This outcome is in line with other demonstrations that PCS at 250% of the currently recommended intake similarly improves multiple measures of metabolic function [58,59,60,64]. This includes an attenuation of fetal overgrowth and postnatal adiposity, improved glucose tolerance and insulin sensitivity, and reduced food intake in mouse and rat models of gestational obesity and high-fat dietary intakes [58,59,60,64,66,74]. This suggests that the current recommendation for choline intake for rodents, at 1g/kg diet, is inadequate for optimal health. Rodent nutrient needs have not been formally reviewed since the last assessment in 1995 by the National Research Council (4th edition, Nutrient Requirements of Laboratory Animals) and the parallel reformulation of AIN-76A in 1993 to AIN-93 [37]. A call has been made to reassess and reformulate the composition of AIN-93 [75]. That prenatal choline intakes at 175% to 250% of current recommendations consistently improve adiposity and glucose tolerance indicates that the rodent choline requirement should be re-evaluated.

## 5. Conclusions

We report that PAE caused metabolic dysfunctions in a sex-dependent manner, worsening glucose tolerance in males and promoting adiposity in females, outcomes that mirror findings in both preclinical studies and in adults who experienced PAE. These phenotypes emerged in later life and may reflect the severity of in utero stress and a subsequent reprogramming of hepatic and pancreatic activities and perhaps appetitive behaviors. These outcomes are suppressed by prenatal choline and indicate that PCS alleviates the in utero stress imposed by PAE, perhaps by increasing the availability of choline-related methylation activities. Unexpectedly, PCS also improved the glucose tolerance and attenuated adiposity in offspring that did not experience PAE, and this suggests that the currently recommended levels of choline in AIN-93G might not be adequate for optimal health. Human pregnancies are frequently choline-inadequate [76] and our findings endorse suggestions [68] that such gestational inadequacies may contribute to the rising chronic disease risk.

## Figures and Tables

**Table 1:** Summary of findings.

	Males		Females	
	Response to PAE	Response to Choline	Response to PAE	Response to Choline
Brain Weight	**=**	**=**	**=**	**↑**
Liver Weight	**↑**	**↓**	=	**↓**
Body Weight	**↑**	**↓**	**↑**	**↓**
Fat Mass	**↑**	**↓**	**↑**	**↓**
Lean Mass	=	=	=	=
Fasting Glucose	**↑**	**↓**	=	**↓**
Glucose Intolerance	**↑**	**↓**	=	**↓** (trend)
Glucose Intolerance Normalized	**↑**	=	=	=

↑, increased response; ↓ decreased response; = no change in response.

## Data Availability

Data are available upon reasonable request to the corresponding authors.

## Abbreviations

The following abbreviations are used in this manuscript:

AUC	Area-under-the-curve
IPGTT	Interperitoneal glucose tolerance test
PAE	Prenatal alcohol exposure
PCS	Prenatal choline supplementation
SAH	S-adenosyl-homocysteine
SAM	S-adenosyl-methionine
T2DM	Type-2 diabetes mellitus
